# A New Diffusion Strategy Using an Epidemic Spreading Model for Encryption

**DOI:** 10.3390/e26090760

**Published:** 2024-09-05

**Authors:** Wei Zhang, Guangdong Zhu, Meng Xing, Jingjing Yang, Hai Yu, Zhiliang Zhu

**Affiliations:** College of Software, Northeastern University, Shenyang 110167, China; zhuguangdongwork@gmail.com (G.Z.); 2010512@stu.neu.edu.cn (M.X.); 2310546@stu.neu.edu.cn (J.Y.); yuhai@mail.neu.edu.cn (H.Y.); zzl@mail.neu.edu.cn (Z.Z.)

**Keywords:** epidemic spreading, SVIR model, encryption, chaos

## Abstract

The diffusion phenomenon that exhibits intrinsic similarities is pervasive in cryptography and natural systems, evident in liquid diffusion, epidemic spread, animal migration, and encryption techniques. In cryptography, bytes are systematically diffused in a sequential manner to encrypt the value of each byte in the plaintext in a linear fashion. In contrast, within an epidemic spreading model, the diffusion process can be represented within a complex, multilayered network, encompassing layers such as familial and social transmission dynamics. Transmission links establish connections both within and between individual layers. It has had a more rapid spread than linear approaches due to the particularization of non-linear transmission. In this study, the novelty of a cryptography diffusion strategy based on an epidemic model is first proposed, in which pixels and their dynamic adjacency are considered as vertices and edges, respectively, within a complex network framework. Subsequently, the encryption process is governed by the Susceptible–Vaccinated–Infected–Recovered (SVIR) model integrated with chaotic dynamics. Simulation results demonstrate that the proposed algorithm exhibits faster encryption speed while effectively resisting brute force, statistical, and differential attacks. Furthermore, it demonstrates strong robustness against noise interference and data loss.

## 1. Introduction

With the rapid advancement of computer and mobile device technologies, a vast amount of digital information—including audio, images, and videos—not only threatens personal privacy but also extends to sensitive biometric data, such as fingerprints, iris scans, and facial contours. Personal biometric information exhibits extensive application in various online security verification scenarios owing to its unique characteristics. However, it can present substantial risks to individual safety and property when multimedia information is compromised or falls out of control [1].

Cryptography offers a viable solution to this issue. In recent years, numerous image encryption algorithms have been proposed by researchers [2,3,4,5], spanning substitution boxes [6,7], Josephus traversal [8], hash functions [9], DNA sequence operations [10,11,12], Boolean networks [13,14], and chaos [15,16]. Chaotic systems show several distinctive characteristics that enhance the potential of image security, including randomness, extreme sensitivity to initial values, and continuous iteration [17]. Consequently, numerous researchers have integrated chaos theory with image encryption techniques to develop more secure and reliable image protection solutions [18,19]. This wide range of schemes enhances the spectrum of options available for fortifying image security. Notably, the unique characteristics of chaotic systems have garnered significant attention, positioning them as a compelling and prominent approach in the field of encryption.

Asgari et al. [20] proposed a one-dimensional chaotic map polynomial combination approach. Initially, a replacement matrix was generated exploiting the proposed pseudo-random number generator, after which the pixels of the image were scrambled according to the corresponding values from this matrix to achieve diffusion effects. Zhou et al. [21] introduced a closed-loop diffusion strategy among blocks, in which the original image is segmented into blocks, ensuring that each subsequent ciphertext block is influenced by its predecessor; subsequently, the first ciphertext block is updated based on the initial and final ciphertext blocks as well as the key block. This closed-loop mechanism enhances the sensitivity of the encryption algorithm but fails to offer sufficient resistance against noise attacks. A DNA complementary cycle mutation strategy has been proposed to enhance the diffusion process, leveraging the increased randomness and complexity derived from various parameter combinations that result in diverse mutations. However, this approach is characterized by slower processing speeds [22].

Moreover, the above algorithms require multiple iterations for key sequence generation due to the distinct phases of permutation and diffusion.

The Coronavirus Disease 19 (COVID-19) outbreak emerged as a global pandemic in December 2019. The genomic similarity between COVID-19 and the SARS COV-1 virus outbreak in 2003 was approximately 82%, with identical receptor cells; however, COVID-19 exhibits higher contagiousness, faster transmission, and increased fatality rates [23]. To strengthen prevention and control measures, researchers across various disciplines have employed data-driven models to forecast the virus’s progression and leveraged these insights to mitigate its spread, in which these models effectively captured the rapid and highly stochastic nature of viral transmission in real-world scenarios. Diffusion phenomena can be conceptualized within epidemic spreading models as multi-layer complex networks encompassing family transmissions and social interactions, among others. These diffusion processes facilitate more rapid dissemination while simultaneously enhancing security due to their non-linear and highly random nature. 

To further ensure the security of the algorithm, Li et al. [24] proposed a chosen-plaintext attack strategy in order to effectively validate the security vulnerability of the image encryption algorithm that presented a novel two-dimensional chaos map proposed in [25]. The outcomes proved that a remarkable image encryption algorithm requires focusing on the randomness of pseudo-random sequence, computational overhead, etc. Additionally, they summarized the challenges in the existing image encryption algorithm, including the following: (1) certain inherent characteristics of some pseudo-random generators facilitate a chosen-plaintext attack; (2) the influence of unique storage format of image data for image quality; codec errors are exhibited in inherent format codec when the data have a mismatched format; (3) the randomness quality of the pseudo-random number is unconsidered; and (4) the computational complexity of the proposed algorithm should be considered. An ideal image encryption method has pervasive application and low computational complexity. Other challenges in image encryption are encapsulated in [26], including the following: (1) S-box is an efficient and ideal method to obscure the relationship of the key and the cipher image, and its widespread use will enhance information security; (2) the lack of convincing security assessment metrics in image encryption schemes; and (3) the previous lessons from cryptanalysis are ignored.

To compensate for the above challenges, the Chen chaotic system is utilized in this study, which is pervasively employed in the field of cryptography, and numerous existing studies have corroborated that the pseudo-random sequence generated by the Chen system enhances robustness against chosen-plaintext attacks [27,28]. Meanwhile, extensive experiments are conducted to justify the randomness and security of the generated keys. There is no format incompatibility issue due to the pixels in the original image being encrypted without compression. To ensure and validate the effectiveness and feasibility of the scheme, we calculated and analyzed the time complexity of the presented methodology which is approximately O(8HW). Furthermore, we propose an epidemic-based diffusion strategy that incorporates a content-adaptive keystream generation scheme to enhance the sensitivity towards plaintexts within our encryption system. In addition, a novel diffusion scheme based on epidemic modeling was introduced that integrates permutation and diffusion operations into a cohesive approach to achieve superior encryption outcomes.

The contributions and novelty of this study can be summarized as follows:

1. A novel approach was introduced that integrates immune concepts into image encryption via an epidemic model combined with complex networks that enhance the randomness of the encryption rules, thereby strengthening the algorithm’s resistance to cryptanalysis and increasing overall security.
2. A novel adaptive keystream generation scheme is proposed to ensure robustness against differential attacks, chosen-plaintext attacks, and known-plaintext attacks.
3. The proposed algorithm exhibited resilience against noise attacks and data loss owing to the independent encryption process applied to each pixel.

The remainder of this paper is structured as follows. Section 2 presents the epidemic model, specifically focusing on the SVIR model employed in this study. Section 3 elaborates on the encryption and decryption processes, including the proposed diffusion strategy, based on the SVIR model. Section 4 assesses the security of the proposed encryption algorithm and demonstrates its feasibility and effectiveness. Finally, Section 5 provides a comprehensive conclusion.

## 2. The Epidemic Model

The establishment of epidemic models dates to the early 20th century. In 1916–1917, Ross et al. conducted an extensive investigation into the frequent spread of epidemics among populations [29,30,31]. Building upon previous research and conclusions, William Ogilvy Kermack and A.G. McKendrick proposed the first model for studying the evolution of infectious diseases in 1927, known as the Susceptible–Infectious–Recovered (SIR) model. Compartmental models are employed in the mathematical modeling of infectious diseases by segmenting populations into distinct compartments, each labeled according to specific states such as susceptible (S), infectious (I), and recovered (R). Individuals transition between these compartments, with the sequence of labels typically indicating the progression of states; for example, an individual’s state transitions from susceptible to infectious and then to recovered in the SIR model.

**Figure 1:**
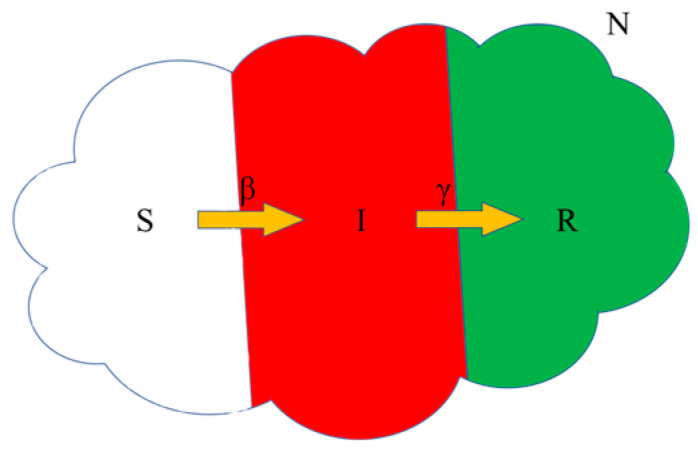
Susceptible–Infectious–Recovered (SIR) model.

The SIR model, illustrated in Figure 1, is regarded as one of the simplest models due to the relatively short time span of infectious disease progression compared to the processes of birth and death. By disregarding the influence of birth and death, the differential equations governing the SIR model can be mathematically expressed as follows:

(1) $$\left\{\begin{array}{l} \frac{dS\left(t\right)}{dt}=-\beta S(t)I(t),\\ \frac{dI\left(t\right)}{dt}=-\beta S(t)I(t)-\gamma I(t),\\ \frac{dR\left(t\right)}{dt}=\gamma I(t), \end{array}\right.$$

where the variables *S*, *I*, and *R* represent the number of susceptible, infected, and recovered individuals, respectively. The parameter *β* denotes the infection rate, while *γ* represents the recovery rate for infected individuals. Additionally, *N* = *S* + *I* + *R* corresponds to the total population size. In 1929, Soper et al. [32] proposed a novel model for the measles virus, which was subsequently expanded by Wilson et al. [33]. Due to the latent phase of the measles virus during transmission, an additional E-state has been incorporated into the SIR model, giving rise to the SEIR model. The E-state represents a fixed incubation period between susceptibility and infection. Instead of transitioning directly from the S state to the I state, individuals enter a distinct incubation period, where they remain for a predetermined duration. They are not transmissible in this stage despite individuals having already contracted the infection.

The basic reproductive number is a crucial parameter in epidemic modeling, representing the expected number of infections caused by a single case in a completely susceptible population without any preventive measures [34]. It can be defined as the ratio of the infection rate to the recovery rate, as shown in Equation (2):

(2) $$R_{0}=\frac{\beta }{\gamma },$$

where *R*_0_ < 1 indicates disease eradication, whereas *R*_0_ > 1 signifies the onset of person-to-person transmission without current containment and treatment measures in place; higher values of *R*_0_ correspond to increased challenges in disease control.

**Figure 2:**
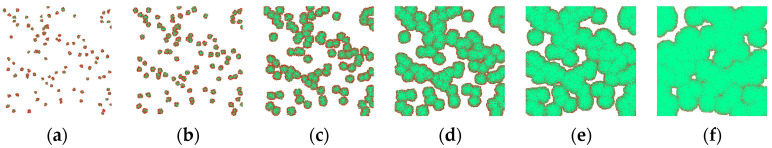
Virus transmission with R_0_ in the SIR model. (**a**–**c**) are R_0_ = 1.062, R_0_ = 1.114, and R_0_ = 1.169, respectively. (**d**–**f**) are R_0_ = 1.023, R_0_ = 0.932, and R_0_ = 0.822, respectively.

We employed white pixels to represent susceptible individuals, red pixels to denote infected individuals, and green pixels to indicate recovered individuals to investigate the impact of *R*_0_ on disease spread. As depicted in Figure 2a–c, there is a sharp increase in the number of currently infected individuals, suggesting that the virus is challenging to control and spreading rapidly. In Figure 2d–f, it is evident that the infection growth rate has significantly slowed, resulting in a marked reduction in the number of infected individuals, thereby indicating successful containment efforts. Consequently, regulating the value of *R*_0_ affects all individual states that are similar to the diffusion processes in image encryption algorithms. It is essential to design more sophisticated propagation models, such as the SVIR model, in order to achieve improved encryption results and further obscure the correlation between adjacent pixels in the ciphertext image.

**Figure 3:**
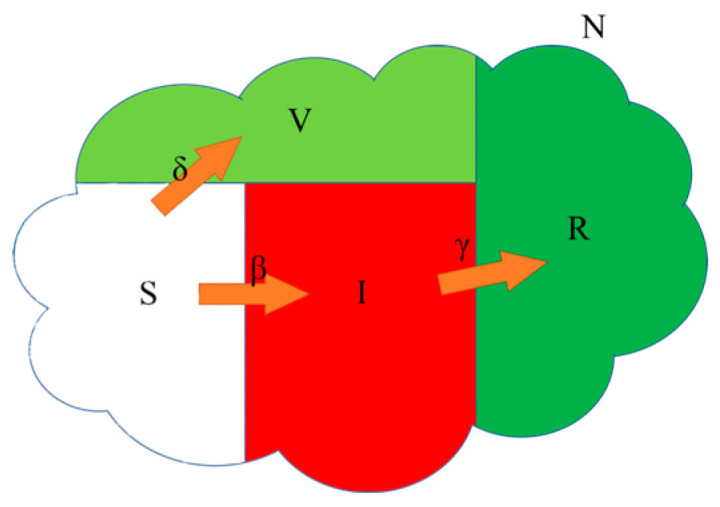
Susceptible–Vaccinated–Infectious–Recovered (SVIR) model.

Vaccination is a highly effective and practical measure for preventing disease spread. Alexander et al. [35] proposed the SVIR model shown in Figure 3, which categorizes a population into four compartments: S (susceptible), V (vaccinated), I (infected), and R (recovery). The SVIR model considers two sources that contribute to an increase in susceptible populations: newborns and a fraction of previously recovered or vaccinated individuals who have lost their immunity over time. Although reinfection is possible among individuals who were previously immune, their susceptibility remains substantially lower compared to that of individuals who are currently susceptible. The SVIR model is computed as follows:

(3) $$\left\{\begin{array}{l} \frac{dS\left(t\right)}{dt}=-\beta S\left(t\right)I\left(t\right)-\rho S\left(t\right)+\lambda R\left(t\right),\\ \frac{dV\left(t\right)}{dt}=\rho S\left(t\right)-\delta V\left(t\right),\\ \frac{dI\left(t\right)}{dt}=\beta S\left(t\right)I\left(t\right)+\delta V\left(t\right)-\gamma I\left(t\right),\\ \frac{dR\left(t\right)}{d\tilde{t}}=\gamma I\left(t\right)-\lambda R\left(t\right), \end{array}\right.$$

where *ρ* ∈ [0.05, 0.3], *δ* ∈ [0.01, 0.05], and *λ* ∈ [0.001, 0.1] are the vaccination rates, vaccine failure rates, and immunity decay rates.

The adjacent pixels’ correlation in the ciphertext image is effectively disrupted by utilizing ‘vaccinated’, thereby bolstering the encryption algorithm’s resistance to differential attacks. Consequently, a novel diffusion strategy is proposed that incorporates the SVIR model into image encryption, which is explained in more detail in the subsequent section.

## 3. The Proposed Method

### 3.1. Encryption Process

The proposed diffusion strategy combines permutation and diffusion into a unified operation, allowing for the direct processing of the natural image that can be defined as follows:

(4) $$I_{C}=SD\left(I_{P},N_{P},Key,\sigma ,\beta ,\delta ,\gamma \right),$$

where the proposed diffusion function *SD*() was utilized, where *I_P_* and *I_C_* denote the plaintext and ciphertext images, respectively. Additionally, *N_P_* represents the pseudo-random number, whereas *σ* = 0.6, *β* = 0.8, *δ* = 0.05, *γ* = 0.5, ρ=0.05, and λ=0.05 correspond to the natural immunity, infection, vaccination, and recovery rates, respectively.

The immune population can be categorized into three groups according to the SVIR model and empirical evidence: individuals with natural immunity, those who have been vaccinated during viral transmission, and those who have recovered from the infection. We concentrated on two principal transmission routes: social and familial. The step-by-step details are outlined as follows.

Step 1: The pixels of the plaintext image are designated as S-state, and the Chen system is iterated to generate the pseudo-random number sequence *N_P_*. 

Step 2: Based on the natural immunity rate σ and a pseudo-random number *N_p_*, *v* pixels with natural immunity are selected and designated as V-state, which is computed in Equation (5):

*v* = ⌊*width* × *height* × *σ*⌋, (5)

where *σ* is 0.6.

Step 3: Typically, the disease originates from a specific location, with pseudo-random numbers employed to select the initial infected pixels.

**Figure 4:**
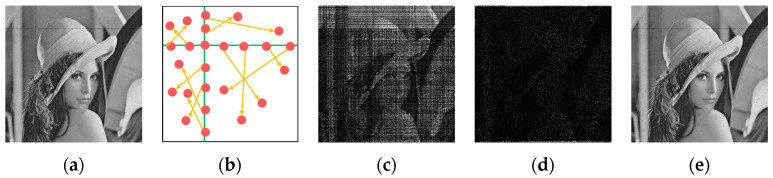
Diffusion strategy based on the SVIR model. (**a**) represents the social network of the first infected pixel, (**b**) represents the correspondence between infection pixels and communities, (**c**) represents the affected area after Step 4, (**d**) represents the result of center permutation, and (**e**) expresses the encrypted result.

Step 4: The row and column of the initially infected pixel are considered as the social network, representing the pathway for virus transmission (Figure 4a). Viral transmission occurs when pixels within the social network reach 50% probability of becoming infected, transitioning from an S to an I state. It is important to emphasize that any alteration in a pixel’s state also leads to a modification of its corresponding pixel value. The modification of pixel value for the original image is defined in Equation (6):

(6) $$P^{\prime }=P⊕N_{P}^{\prime },$$

where the pixels of the plaintext and the ciphertext images are represented by *P* and *P*′, respectively. The value of *N*′*_p_* is calculated as (*C_pixel_* × *N*_p_) mod 256, where *C_pixel_* denotes the sum of pixels in the plaintext image, which is considered one of the key factors. 

Step 5: The social network of the initially infected pixel partitions the entire image into four segments representing four distinct ‘communities’. The infected pixels within the social network are then associated with their corresponding positions in the respective ‘communities’, as illustrated in Figure 4b, and the relationship is described as follows:

(7) $$\left\{\begin{array}{l} i=p_{i}+(N_{p}\times w\prime ),\\ j=p_{j}+(N_{p}\times h\prime ),\\ f[i][j]=S, \end{array}\right.$$

where (*p_i_*, *p_j_*) denotes the upper-left coordinates of the current ‘community’, ‘*w*′ and ‘*h*′ represent the width and height of the current ’community’, respectively, while *f* [*i*][*j*] is an array applied to indicate the state of the pixel at position (*i*, *j*). 

Step 6: The vaccination operation is implemented to further disrupt the adjacent pixel correlation. A subset of pixels in the S-state is randomly selected and assigned to the vaccination state (I-state). Considering the early stages of virus transmission, a vaccination rate of *δ* = 0.05 was applied.

Step 7: The virus propagates through their social networks and familial connections after the infected pixels have been mapped to their respective locations. The conditions for social transmission remain unchanged, whereas family transmission occurs when an infected pixel infects up to eight adjacent pixels. Furthermore, an increased number of infected pixels surrounding susceptible pixels results in a strengthened infection rate, which is determined by Equation (8):

(8) $$\gamma^{\prime }=\gamma \times \left(1+N_{i}\times 0.1\right),$$

where *γ* = 0.5 is the basic infection rate, and *N_i_* represents the number of infected pixels in the eight neighboring pixels surrounding the current pixel. The extent of infection currently is illustrated in Figure 4c.

Step 8: The main object of the image is typically positioned in the central area. Therefore, we propose employing the “central spread” method:

- First, it is necessary to quantify the number of newly infected pixels generated in Step 2 as *X* while quantifying the number of remaining susceptible pixels as *Y*.
- Next, identify the *Y*/2-th susceptible individual and subsequently observe *n* = *min*(*X*, *Y*) cycles of alternating viral spread on both sides of the *Y*/2-th susceptible individual, in accordance with familial transmission conditions.

The simulation results demonstrate that this operation significantly reduces processing time while achieving a superior diffusion effect. The extent of the infection at this time is shown in Figure 4d. 

Step 9: Distinct recovery rates are assigned to pixels infected at different stages (wherein the pixel state transitions from I to R) to enhance the effectiveness of the algorithm against differential attacks: infected pixels generated in Step 2 exhibit a recovery rate of 80%; the remaining infected pixels demonstrate a recovery rate of 60%.

Step 10: During the diffusion phase, the pixels of the original image are stored based on their infection order, and the remaining pixels in states ‘S’ and ‘V’ are arranged according to their respective positions. Subsequently, Equation (6) is applied to process the entire ciphertext image, and the final encrypted representation is generated.

### 3.2. Decryption Process

The decryption process is the inverse operation of encryption. Firstly, the complete pseudo-random sequence must be obtained, followed by performing the inverse operation based on Equation (6) to restore the image before pixel value normalization. The corresponding positional relationship for pixel position scrambling is obtained during the acquisition of the pseudo-random sequence. Pixels can be restored to their original positions while retrieving their original gray values by positional relationship, thereby enabling retrieval of the plaintext image.

### 3.3. Content-Adaptive Keystream Generation Scheme

**Figure 5:**
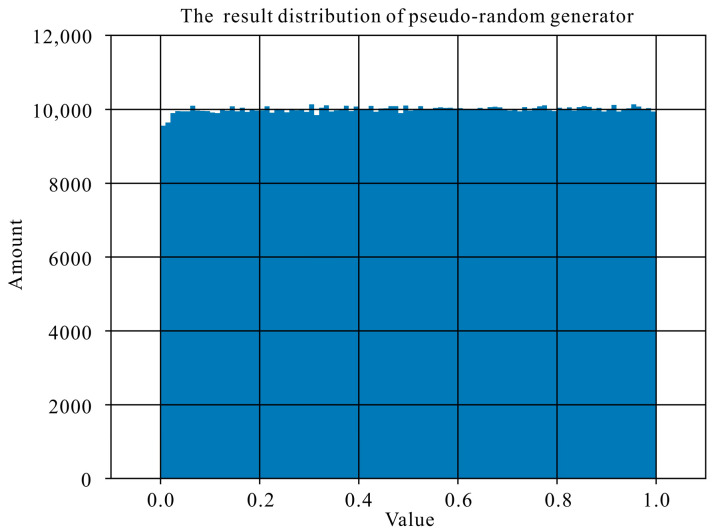
Distribution histogram of the output of the pseudo-random generator 106 times.

Any pseudo-random number generator can be utilized in the proposed algorithm; we illustrate the Chen system [27,28] as an example, wherein initial conditions are set to 0.123456789, 0.567891234, and 0.512312412, individually, and the control parameters are 35, 3, and 28. The keys of the Chen system are denoted as *Key*_1_, *Key*_2_, and *Key*_3_. We ensure a uniform distribution of pseudo-random numbers within this interval by guaranteeing the range of *Key*_1_, whose value is within the range of [0,1], as depicted in Figure 5.

The sum of pixel values in the raw image is incorporated as one of the keys to control the pseudo-random number generator, as detailed below:

*Key*_1_ = *Key*_1_ × *C_pixel_* − ⌊ *Key*_1_ × *C_pixel_* ⌋, (9)

where *C_pixel_* is the sum of the pixel gray value of the plaintext image.

The proposed algorithm ensures that even slight differences between the plaintext images result in completely distinct ciphertext images that effectively withstand the chosen-plaintext attacks.

## 4. Experience and Result

### 4.1. Key Space Analysis

The key space is the set of all available keys and increases exponentially with the length of the key. To effectively resist brute force attacks [36], the key space must exceed 2^112^. In this study, the keys consisted of the initial values of a chaotic system (*Key*_1_, *Key*_2_, and *Key*_3_), which were represented as double precision numbers with a computational precision of 10^12^. Additionally, the sum of the pixels in the plaintext image (*C_pixel_*) and the number of chaotic system preprocessing steps (*N_pre_*) are considered. Therefore, the key space can be estimated as *C_pixel_* × *N_pre_* × *Key*_1_ × *Key*_2_ × *Key*_3_ = *C_pixel_* × *N_pre_* × 10^36^ ≈ *C_pixel_* × *N_pre_* × 2^120^. It is evident that the proposed algorithm provides a sufficiently large key space to withstand violent attacks.

### 4.2. Statistical Attack Analysis

**Figure 6:**
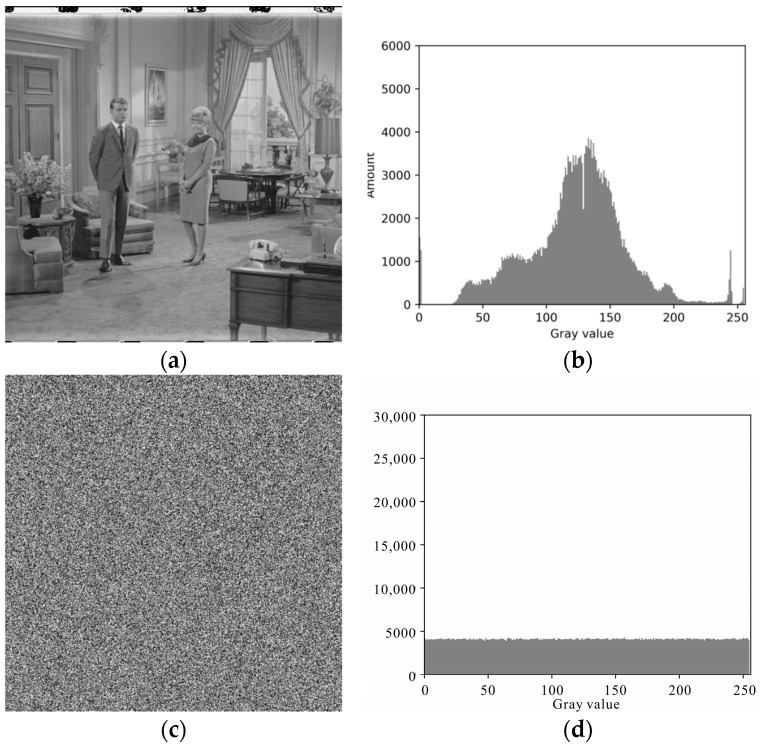

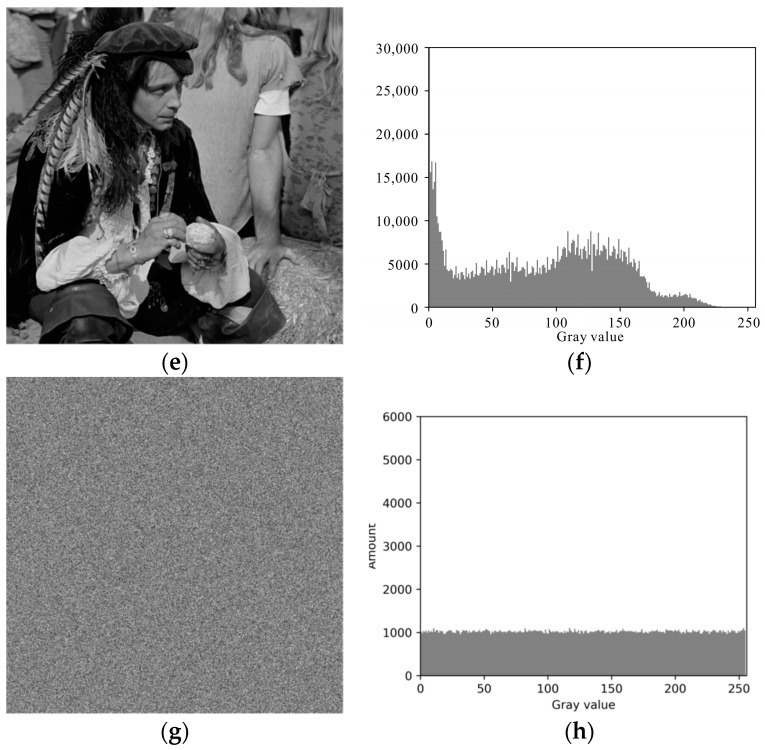
Histogram analysis. (**a**) Image 5.2.08; (**b**) histogram of (**a**); (**c**) encrypted 5.2.08; (**d**) histogram of (**c**); (**e**) image 5.3.01; (**f**) histogram of (**e**); (**g**) encrypted 5.3.01; (**h**) histogram of (**g**).

The histogram illustrates the distribution of pixel values within the images. Typically, visually meaningful images exhibit a regular histogram distribution, whereas ciphertext images show an evenly distributed histogram to prevent statistical attacks. Figure 6 illustrates the histograms of the two USC-SIPI database [37] images and their corresponding ciphertext versions (Figure 6). All ciphertext image histograms display uniform distribution and visual similarity, rendering it challenging for potential attackers to extract valuable information through statistical analysis.

We employed a chi-square test to further validate the uniformity of the gray value distribution in the ciphertext images:

(10) $$\chi^{2}=\sum_{i=1}^{255}\frac{f_{i}-f_{e}}{f_{e}},$$

where the significance level was set to 0.05, and the test results for multiple images of different sizes are presented in Table 1, where $f_{e}=\frac{M\times N}{255}$, and *f_i_* denotes the number of pixels in the cipher images with a pixel value of *i*. It can be observed that all ciphertext images obtained by the proposed algorithm have values lower than $\chi_{0.05}^{2}$ (255) = 293.247, indicating superior performance compared to [38,39,40,41].

### 4.3. Correlated Analysis

**Figure 7:**
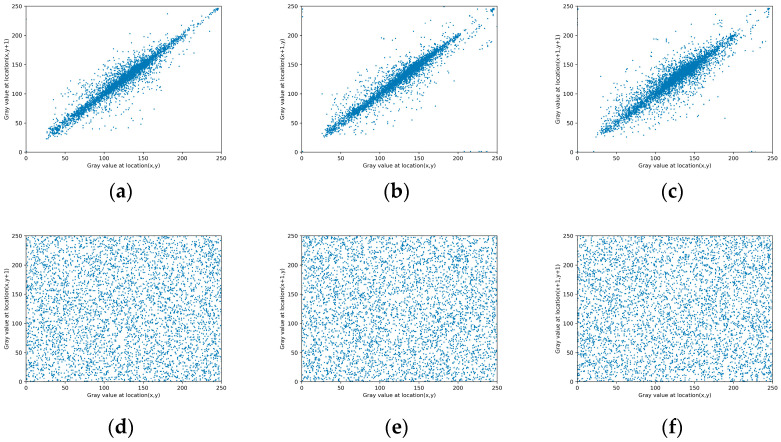
Correlation analysis of the adjacent two pixel for image 5.2.08 (couple). (**a**,**d**) Horizontal correlation of plaintext and ciphertext images; (**b**,**e**) vertical correlation of plaintext and ciphertext images; (**c**,**f**) diagonal correlation of plaintext and ciphertext images.

A plaintext image exhibits a strong correlation between adjacent pixels, whereas a ciphertext image demonstrates a significantly weakened correlation due to sufficient scrambling of the pixels [42]. Three thousand pairs of randomly selected adjacent pixels were extracted from image 5.2.08 (couple) to assess the correlation between the encrypted image pixels using the algorithm proposed in this paper. Figure 7 illustrates the correlation among adjacent pixels in image 5.2.08 (couple), which corresponds to encrypted images in the horizontal, vertical, and diagonal directions.

The correlation coefficient between adjacent pixels is calculated as follows:

(11) $$\left\{\begin{array}{l} r_{xy}=\frac{\mathrm{cov}(u,v)}{\sqrt{D\left(u\right)}\sqrt{D\left(v\right)}},\\ \mathrm{cov}(u,v)=\sum_{i=1}^{N}\frac{\left(u_{i}-E\left(u\right)\right)\left(v_{i}-E\left(v\right)\right)}{N},\\ D(u)=\sum_{i=1}^{N}\frac{(u_{i}-E(u){)}^{2}}{N},\\ E(u)=\sum_{i=1}^{N}\frac{u_{i}}{N}, \end{array}\right.$$

where *u* and *v* represent the gray values of two adjacent regions; *E*(*u*) and *D*(*u*) are the mean and variance values, respectively; and *cov*(*u*, *v*) represents the covariance. Table 2 demonstrates that the adjacent pixels’ correlation in encrypted images is extremely low and indicates the algorithm’s effective diffusion properties. 

### 4.4. Information Entropy Analysis

Information entropy quantifies the uncertainty inherent in image data, with higher entropy reflecting greater levels of uncertainty that is formally defined as follows:

(12) $$H\left(I\right)=-\sum_{i=1}^{N}p\left(u_{i}\right)\log_{2}p\left(u_{i}\right),$$

where *u_i_* is the *i*-th gray value, and *p*(*u_i_*) is the probability of gray level *u_i_*. The theoretical value of the information entropy is *H*(*I*) = 8. The closeness of the information entropy of an image to the theoretical value indicates that the employed encryption algorithm offers a higher level of security against entropy attacks [43]. Information entropies of different images encrypted using various methods are listed in Table 3. As shown in Table 3, the proposed algorithm yields ciphertext images with information entropies that closely align with the theoretical value of eight, thereby confirming their high randomness. Consequently, we can conclude that the proposed algorithm is robust to entropy attacks.

### 4.5. Differential Attack Analysis

A differential attack is a chosen-plaintext attack that targets encryption algorithms by analyzing the impact of specific differences in plaintext on the resulting ciphertext [44]. To crack the key, attackers typically introduce subtle modifications to the plaintext image and then compare the ciphertext images before and after these alterations to extract key information. Consequently, a secure encryption algorithm should generate significantly distinct ciphertexts even with minor changes in the corresponding plaintext.

The resist differential attack performance of the encryption algorithm was evaluated by selecting several images from the SIPI database and generating new images exploiting random pixel value changes [45]. The difference between the corresponding ciphertext images was measured utilizing the Number of Changing Pixel Rate (NPCR) and Unified Averaged Changed Intensity (UACI) that are defined as follows:

(13) $$NPCR=\sum_{i=1}^{M}\sum_{j=1}^{N}\frac{D\left(i,j\right)}{M\times N}\times 100\%,$$

(14) $$UACI=\sum_{i=1}^{M}\sum_{j=1}^{N}\frac{\left|c_{1}\left(i,j\right)-c_{2}\left(i,j\right)\right|}{255\times M\times N}\times 100\%,$$

(15) $$D\left(i,j\right)=\left\{\begin{array}{l} 0,\;if\;c_{1}(i,j)=c_{2}(i,j);\\ 1,\;otherwise. \end{array}\right.,$$

where *M* and *N* are the width and height of the image, respectively, and *c*_1_ and *c*_2_ are ciphertext images corresponding to two plaintext images with one pixel difference.

Wang et al. [46] proposed critical values of the NPCR and UACI at different significance levels, which enabled a more accurate evaluation of the resistance of the image encryption algorithm against differential attacks. The NPCR and the UACI critical values for gray images of different sizes under varying significance levels are listed in Table 4 and Table 5, respectively. As shown in Table 6, the proposed encryption algorithm successfully passed the NPCR and UACI tests across all tested images. These results confirm that our encryption algorithm exhibits excellent resilience against differential attacks.

### 4.6. Key Sensitivity Analysis

**Figure 8:**
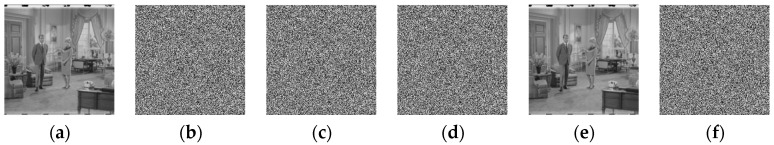
Key sensitive analysis. (**a**) The original image; (**b**) encryption; (**a**) with key K; (**c**) encryption of (**a**) with key K′; (**d**) the different between them (**b**,**c**) (calculated by using |(**c**,**d**)|); (**e**) decryption (**b**) with key K; (**f**) decryption (**b**) with key K′.

The sensitivity of a secure encryption algorithm to the key is crucial so that completely different encryption and decryption outcomes are rendered due to a slight modification of the key [47]. As depicted in Figure 8, this study demonstrates the algorithm’s remarkable key sensitivity. Any alteration that affects only the random bits within the random key, while leaving other components unchanged (as shown in Table 7), makes it impossible to decrypt the ciphertext image. Experimental results confirm that this algorithm exhibits excellent key sensitivity.

### 4.7. Robustness Analysis

**Figure 9:**
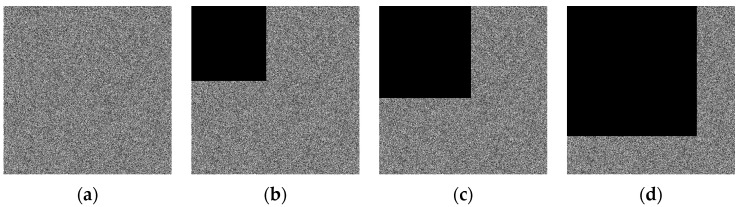

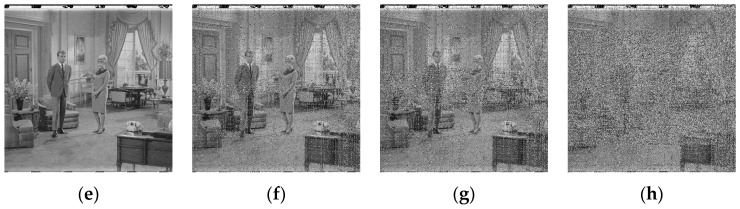
Robustness analysis. (**a**–**d**) represent the encrypted image with 20% salt-and-pepper noise, 20%, 30%, and 60% black blocks, and corresponding recovered image are exhibited in (**e**–**h**), respectively.

Data loss or tampering can occur easily during digital image transmission and storage, leading to inaccurate data that can affect the image decryption quality [48]. A robust encryption algorithm must withstand pixel changes. We introduced 20% salt-and-pepper noise and black blocks representing 20%, 30%, and 60% lost data in order to evaluate the performance of the proposed algorithm against noise interference and data loss. The corresponding recovered images are presented in Figure 9. As shown in Figure 9, although the image quality gradually deteriorates with increasing noise and data loss ratios after decryption, it remains visually recognizable, indicating that the proposed algorithm exhibits good robustness.

### 4.8. Speed Analysis

The efficiency of the encryption algorithms is of paramount importance in practical applications [49]. The time complexity of the proposed method is approximately O(8HW). Furthermore, we conducted a comprehensive analysis and comparison of the encryption time and the proposed algorithm under identical hardware (AMD Ryzen 5 3600, 16 GB memory, NVIDIA RTX 2070 8 GB) and software conditions (Visual Studio 2019 in Windows 10 operating system). The different images are tested to obtain average encryption times, as presented in Table 8. Comparative results demonstrate that the proposed algorithm achieves comparable or superior encryption effects within a shorter timeframe.

## 5. Conclusions

An image encryption algorithm based on the epidemic model is presented, characterized by rapid propagation and extensive coverage, designed to cater to the real-time encryption demands of modern society. The correlation between pixels in the ciphertext image is further disrupted by incorporating natural immunity and vaccination. Simulation results demonstrate that this algorithm exhibits strong robustness against noise, differential, and statistical attacks while performing in real time compared to existing methods. A notable drawback is the increased processing time needed to achieve more complex propagation processes and enhanced encryption effects. In the future, our work will focus on reducing the processing time.

## Figures and Tables

**Table 1:** Chi-square test for the plaintext image and ciphertext image.

Test Image	Size	Plaintext Image	Proposed	[37]	[38]	[39]	[40]
4.1.04	256 × 256	68,976	235.9843	236.1250	248.9609	270.2343	**226.4765**
4.1.05	256 × 256	301,008	**225.9453**	248.3203	264.5468	246.6406	268.8046
4.1.06	256 × 256	66,014	243.8515	**207.3515**	265.1718	272.2812	227.6640
5.1.09	256 × 256	135,687	**227.4843**	271.5078	252.8828	290.8125	234.7500
5.1.10	256 × 256	50,862	258.4843	265.9921	257.6562	**205.0703**	255.2656
5.1.11	512 × 512	220,848	**213.0390**	251.6953	260.5390	270.1796	255.2656
5.2.08	512 × 512	298,865	**178.4707**	255.1777	252.5312	260.9433	281.2929
5.2.09	512 × 512	441,857	**228.5078**	288.6875	273.5761	226.4355	229.1171
5.2.10	512 × 512	1,185,618	**226.8476**	232.0996	230.6562	278.5878	233.8203
7.1.08	512 × 512	2,887,769	**233.6972**	252.8554	249.7597	279.5761	315.3652
7.1.09	512 × 512	957,952	**233.5859**	260.6835	263.4335	261.4960	254.1562
7.1.10	512 × 512	1,194,451	**246.5253**	265.6718	270.6816	267.8281	259.9785
5.3.01	1024 × 1024	709,340	**244.5791**	252.6191	260.0273	269.1308	257.1904
5.3.02	1024 × 1024	1,974,776	**232.6528**	260.1171	237.1645	301.1362	255.0214
7.2.01	1024 × 1024	7,199,928	**253.9165**	270.0195	260.5532	275.8862	256.3408

**Table 2:** The correlation among adjacent pixels for different images utilizing distinct methods.

Image	Size	Horizontal				Vertical				Diagonal			
		Plaintext Image	Ciphertext Image			Plaintext Image	Ciphertext Image			Plaintext Image	Ciphertext Image		
			Proposed	[38]	[39]		Proposed	[38]	[39]		Proposed	[38]	[39]
4.1.04	256 × 256	0.98307	**0.00025**	0.00499	−0.00902	0.96842	**−0.00039**	−0.00056	0.00068	0.95878	**−0.00041**	0.01642	0.00888
4.1.05	256 × 256	0.95536	**−0.00019**	0.00604	−0.00895	0.97736	**−0.00031**	−0.00682	0.00573	0.93546	**−0.00028**	−0.01454	0.00486
4.1.06	256 × 256	0.94710	**−0.00020**	0.03631	0.00855	0.96797	**0.00038**	0.00276	0.00049	0.93356	**0.00029**	−0.00390	−0.00877
5.1.09	256 × 256	0.93517	**0.00042**	−0.00485	−0.00291	0.90198	**0.00018**	0.00032	0.02024	0.90522	−0.00052	0.01426	**0.00045**
5.1.10	256 × 256	0.85464	**−0.00098**	−0.01990	**0.00098**	0.90556	−0.00081	0.00919	**0.00064**	0.81217	**0.00057**	−0.00651	−0.02706
5.1.11	512 × 512	0.94800	**0.00040**	−0.00328	0.02324	0.95562	**0.00032**	−0.03026	0.00716	0.88937	**−0.00080**	0.00520	0.00629
5.2.08	512 × 512	0.86386	**0.00043**	0.00917	−0.00064	0.95508	**−0.00075**	−0.00645	−0.00173	0.82131	**0.00011**	−0.01403	0.00797
5.2.09	512 × 512	0.86685	**0.00039**	−0.01860	−0.00230	0.89618	−0.00063	**0.00040**	−0.01985	0.80771	**0.00023**	−0.00387	−0.00065
5.2.10	512 × 512	0.93132	**−0.00029**	0.00080	−0.00398	0.94078	**−0.00055**	−0.01831	0.00064	0.90028	**−0.00065**	−0.00694	−0.00528
7.1.08	512 × 512	0.92244	**0.00056**	−0.00087	−0.01085	0.95787	**0.00013**	−0.00124	0.00518	0.92171	**−0.00077**	0.00813	0.00207
7.1.09	512 × 512	0.92773	**−0.00036**	−0.02247	0.00165	0.96471	**0.00182**	0.00992	0.00938	0.91626	**0.00048**	0.00131	0.00235
7.1.10	512 × 512	0.94765	**0.00032**	0.00473	−0.00352	0.96561	−0.00057	**0.00039**	0.00154	0.93088	**0.00085**	0.03198	−0.00359
5.3.01	1024 × 1024	0.98276	**0.00043**	0.00788	−0.00489	0.97701	**0.00014**	−0.00334	0.00488	0.96584	**0.00083**	0.00691	−0.00181
5.3.02	1024 × 1024	0.90675	**0.00063**	0.00840	−0.00714	0.91013	**0.00040**	0.00386	−0.00013	0.86889	**−0.00027**	−0.00285	0.00870
7.2.01	1024 × 1024	0.94948	**0.00037**	0.00225	−0.01367	0.96461	**0.00061**	−0.00172	0.00069	0.94792	**−0.00493**	−0.00289	−0.00216

**Table 3:** Information entropy analysis.

Test Image	Size	Plaintext Image	Proposed	[38]	[39]	[40]	[41]
4.1.04	256 × 256	7.257481	**7.997405**	7.997404	7.997266	7.997027	7.997403
4.1.05	256 × 256	6.496273	**7.997512**	7.997252	7.997086	7.997287	7.997039
4.1.06	256 × 256	7.310220	7.997318	**7.997722**	7.997059	7.996997	7.997484
5.1.09	256 × 256	6.709312	**7.997502**	7.997026	7.997203	7.996785	7.997404
5.1.10	256 × 256	7.311807	7.997154	7.997066	7.997076	**7.997733**	7.997167
5.1.11	512 × 512	6.452275	**7.997653**	7.997220	7.997123	7.997019	7.997185
5.2.08	512 × 512	7.201007	**7.999509**	7.999295	7.999303	7.999279	7.999223
5.2.09	512 × 512	6.993994	**7.999371**	7.999206	7.999247	7.999368	7.999370
5.2.10	512 × 512	5.705560	7.999375	7.999360	7.999363	7.999232	**7.999383**
7.1.08	512 × 512	5.053447	**7.999356**	7.999302	7.999310	7.999229	7.999132
7.1.09	512 × 512	6.189813	**7.999356**	7.999283	7.999275	7.999279	7.999300
7.1.10	512 × 512	5.908789	**7.999319**	7.999267	7.999254	7.999262	7.999287
5.3.01	1024 × 1024	7.523736	**7.999831**	7.999826	7.999821	7.999814	7.999822
5.3.02	1024 × 1024	6.830329	**7.999849**	7.999821	7.999836	7.999792	7.999824
7.2.01	1024 × 1024	5.641453	**7.999834**	7.999814	7.999820	7.999810	7.999823

**Table 4:** Theoretical NPCR critical values for different image sizes at different significance levels.

Image Size	$N_{0.05}^{*}$	$N_{0.01}^{*}$	$N_{0.001}^{*}$
256 × 256	99.5693%	99.5527%	99.5341%
512 × 512	99.5893%	99.5810%	99.5717%
1024 × 1024	99.5994%	99.5994%	99.5906%

**Table 5:** Theoretical UACI critical values for different image sizes at different significance levels.

Image Size	$N_{0.05}^{*}$	$N_{0.01}^{*}$	$N_{0.001}^{*}$
256 × 256	$U_{0.05}^{*-}=33.2824\%$	$U_{0.01}^{*-}=33.2255\%$	$U_{0.001}^{*-}=33.1594\%$
	$U_{0.05}^{*+}=33.6447\%$	$U_{0.01}^{*+}=33.7016\%$	$U_{0.001}^{*+}=33.7677\%$
512 × 512	$U_{0.05}^{*-}=33.3730\%$	$U_{0.01}^{*-}=33.3445\%$	$U_{0.001}^{*-}=33.3115\%$
	$U_{0.05}^{*+}=33.5541\%$	$U_{0.01}^{*+}=33.5826\%$	$U_{0.001}^{*+}=33.6156\%$
1024 × 1024	$U_{0.05}^{*-}=33.4183\%$	$U_{0.01}^{*-}=33.4040\%$	$U_{0.001}^{*-}=33.3875\%$
	$U_{0.05}^{*+}=33.5088\%$	$U_{0.01}^{*+}=33.5231\%$	$U_{0.001}^{*+}=33.5396\%$

**Table 6:** The result of the critical test for NPCR and UACI.

Test Image	Size	NPCR Score	NPCR Critical Test Result	UACI Score	UACI Critical Test Result
4.1.04	256 × 256	99.6292	Pass	33.6310	Pass
4.1.05	256 × 256	99.5941	Pass	33.4498	Pass
4.1.06	256 × 256	99.6246	Pass	33.4227	Pass
5.1.09	256 × 256	99.5834	Pass	33.4848	Pass
5.1.10	256 × 256	99.6017	Pass	33.2992	Pass
5.1.11	512 × 512	99.6017	Pass	33.3886	Pass
5.2.08	512 × 512	99.6139	Pass	33.5141	Pass
5.2.09	512 × 512	99.6025	Pass	33.5387	Pass
5.2.10	512 × 512	99.6204	Pass	33.5026	Pass
7.1.08	512 × 512	99.6120	Pass	33.4629	Pass
7.1.09	512 × 512	99.6356	Pass	33.5319	Pass
7.1.10	512 × 512	99.6009	Pass	33.4521	Pass
5.3.01	1024 × 1024	99.6007	Pass	33.4597	Pass
5.3.02	1024 × 1024	99.6182	Pass	33.4626	Pass
7.2.01	1024 × 1024	99.6001	Pass	33.4672	Pass

**Table 7:** Key sensitivity analysis (‘-’ indicates that the key is not changed).

Key	*K*	*K*′
*Key*_1_	0.123687176893	-
*Key*_2_	0.896876889082	-
*Key*_3_	0.676145256343	0.676245256342
*C_piexl_*	32290163	-
*N_pre_*	1000	-

**Table 8:** Encryption times for different images (unit: s).

Image Size	Proposed	[37]	[38]	[39]	[40]
256 × 256	**0.024**	0.030	0.097	0.210	0.1275
512 × 512	**0.080**	0.102	0.253	0.689	0.5316
1024 × 1024	**0.318**	0.403	0.762	2.875	2.4613

## Data Availability

The data presented in this study are available on request from the corresponding author.
